# Facet-controlled growth and soft-chemical exfoliation of two-dimensional titanium dioxide nanosheets

**DOI:** 10.1039/d4na00442f

**Published:** 2024-07-16

**Authors:** Christian Harito, Munawar Khalil, Leanddas Nurdiwijayanto, Ni Luh Wulan Septiani, Syauqi Abdurrahman Abrori, Budi Riza Putra, Syed Z. J. Zaidi, Takaaki Taniguchi, Brian Yuliarto, Frank C. Walsh

**Affiliations:** a Industrial Engineering Department, BINUS Graduate Program – Master of Industrial Engineering, Bina Nusantara University Jakarta Indonesia christian.harito@binus.ac.id; b Department of Chemistry, Faculty of Mathematics and Natural Sciences, Universitas Indonesia Kampus Baru UI Depok Jawa Barat Indonesia; c Research Center for Materials Nanoarchitectonics (MANA), National Institute for Materials Science (NIMS) 1-1 Namiki Tsukuba Ibaraki 305-0044 Japan; d Research Center for Advanced Materials, National Research and Innovation Agency Komplek PUSPIPTEK, Serpong South Tangerang 15314 Banten Indonesia; e Automotive & Robotics Program, Computer Engineering Department, BINUS ASO School of Engineering, Bina Nusantara University Jakarta 11480 Indonesia; f Research Center for Metallurgy, National Research and Innovation Agency (BRIN) PUSPIPTEK Area, Building No. 470, Setu Regency South Tangerang Banten 15314 Indonesia; g Institute of Chemical Engineering and Technology, University of the Punjab Lahore Pakistan; h Department of Engineering Physics, Advanced Functional Materials Laboratory, Institute of Technology Bandung (ITB) Bandung 40132 Indonesia; i Research Center for Nanosciences and Nanotechnology (RCNN), Institute of Technology Bandung (ITB) Bandung 40132 Indonesia; j Electrochemical Engineering Laboratory, Faculty of Engineering and Physical Sciences, University of Southampton Southampton UK

## Abstract

TiO_2_ remains one of the most popular materials used in catalysts, photovoltaics, coatings, and electronics due to its abundance, chemical stability, and excellent catalytic properties. The tailoring of the TiO_2_ structure into two-dimensional nanosheets prompted the successful isolation of graphene and MXenes. In this review, facet-controlled TiO_2_ and monolayer titanate are outlined, covering their synthesis route and formation mechanism. The reactive facet of TiO_2_ is usually controlled by a capping agent. In contrast, the monolayer titanate is achieved by ion-exchange and delamination of layered titanates. Each route leads to 2D structures with unique physical and chemical properties, which expands its utilisation into several niche applications. We elaborate the detailed outlook for the future use and research studies of facet-controlled TiO_2_ and monolayer titanates. Advantages and disadvantages of both structures are provided, along with suggested applications for each type of 2D TiO_2_ nanosheets.

## Abstract 2

A timely review covering facet-controlled growth and soft-chemical exfoliation of two-dimensional titanium dioxide nanosheets.

## Graphical abstract

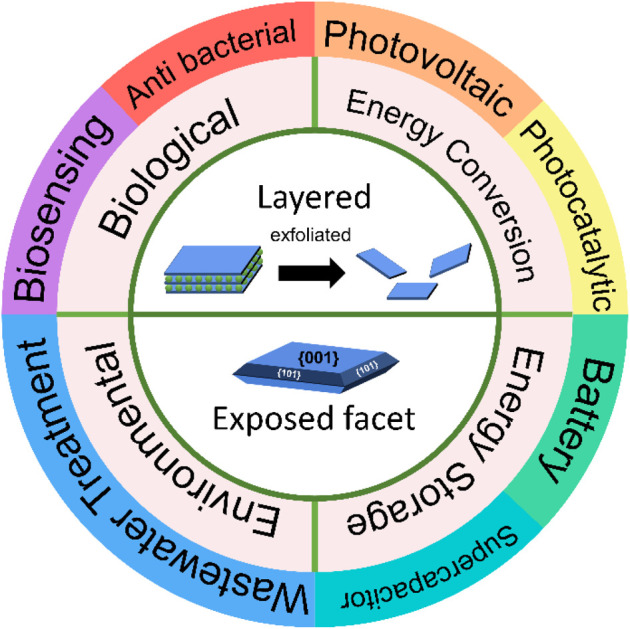

## 1. Introduction

Titanium, as the 9th most abundant element in the Earth's crust, is naturally found in the form of oxide minerals, particularly titania, TiO_2_. Over the last fifty years, TiO_2_ has been utilised in many applications involving photocatalysts, photovoltaics, corrosion/UV protection coatings, and electronics while further studies exploring novel uses continue. Modification of the titanium oxide morphology into tailored nanostructures is sought by many practitioners since it is able to amplify functionality due to a larger active surface area, leading to higher reactivity. Many unique properties can only be observed at the nanoscale regime. For instance, quantum confinement may occur at a nanoscale thickness, tuning in the density of states and band gap of nanomaterials.^1^ In catalysis, the exposed facets (surface orientation) of nanomaterials play a crucial role. Certain facets may have higher catalytic activity due to their crystallographic orientation, making them more effective in promoting chemical reactions. For titania, quantum confinement and surface orientation play a major role in photoconversion efficiency.^2^

Since the rise of graphene over the last two decades,^3^ the promise of this unique material has accelerated research interest in inorganic 2D nanomaterials. The rapid development of 2D nanomaterials is not limited to carbonaceous materials. Recently, titanium carbide-based 2D nanosheets, known as MXenes, have received much attention. Since 2011, an article on the exfoliation of MXenes (*i.e.*, Ti_3_AlC_2_) by HF has received over 2500 citations,^4^ indicating the rapid growth of research. Titanium oxide nanosheets, a 2D analogue of MXenes, have also shown an academic impact, especially in catalysis; a 2008 contribution on anatase TiO_2_ with exposed facets has been cited over 3000 times.^5^ Titania itself has enjoyed a huge impact and has helped transform our knowledge of photoelectrochemical cells since 1972.^6^ Titanium oxide-based nanosheets are an important research topic, which merits a comprehensive review to update our fundamental knowledge and awareness of their uses.

**Fig. 1:**
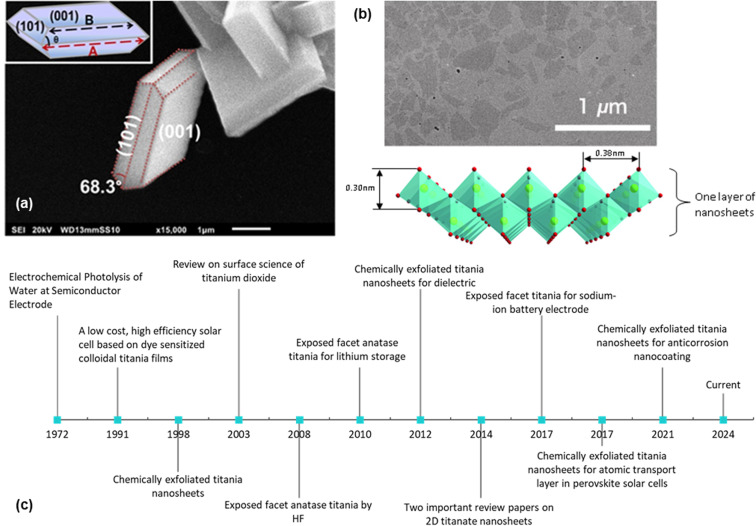
(a) SEM image of the exposed {001} facet of TiO_2_ with illustration (inset);^13^ (b) TEM image of the chemically exfoliated titania nanosheets and its 2D structure;^14^ (c) historical timeline from the ground-breaking classic research on TiO_2_ (ref. 6 and 15) to the development of the exposed facet TiO_2_ (ref. 5, 10–13 and 16) and single-layered titania nanosheets.^8,17–19^ Panel (a) is adapted with permission.^13^ Copyright © 2017 American Chemical Society. Panel (b) is adapted with permission from John Wiley and Sons.^14^ Copyright © 2010 WILEY-VCH.

2D nanosheets typically have a thickness of a few nanometres. They can be divided into three categories, namely, exposed facet TiO_2_ nanosheets, multi-layered nanosheets and monolayered nanosheets. Exposed facet TiO_2_ nanosheets are thin non-layered materials with a 3D crystallographic structure (*i.e.*, TiO_2_ with dominant {001} facets), as shown in Fig. 1a. The thickness of this type of nanosheets could reach <5 nm while maintaining the crystallographic structure of TiO_2_.^7^ Meanwhile, layered titanium oxide nanosheets consist of thin layer structures made from TiO_6_ octahedra, as shown in Fig. 1b. Monolayered or single-layered titanium oxide nanosheets has been extensively researched by Sasaki *et al.*^8^ who discovered a two-step method to exfoliate titanium oxide (titanate) nanosheets in 1998. In contrast to research on exposed facets TiO_2_, which mainly focuses on photocatalysis, research on the monolayered titanate nanosheets extends the exploration of their functionalities/properties, such as dielectric characteristics, together with spin-electronic applications.^9^ While titanium oxide nanosheets have potential in electrochemistry,^10–12^ applications remain exploratory.

2D TiO_2_ nanosheets also offer distinct advantages over other 2D materials primarily due to their exceptional chemical stability and abundant availability. For example, MXenes, while promising for various applications, often suffer from oxidation and stability issues, limiting their long-term usability in harsh environments. In contrast, TiO_2_ nanosheets are highly resistant to chemical degradation, ensuring consistent performance over time. Meanwhile, although graphene is renowned for its exceptional electrical conductivity and mechanical strength, it often lacks the inherent photocatalytic properties of TiO_2_ nanosheets. This makes them less suitable for environmental remediation and energy conversion applications, such as photocatalytic water splitting and pollutant degradation. Besides, producing high-quality graphene can be expensive and challenging to scale up, whereas TiO_2_ nanosheets are more cost-effective and accessible in large quantities.

Considering their advantages, this review aims to offer a comprehensive perspective on both the synthesis techniques and the distinct material properties of two key types: exposed facet TiO_2_ nanosheets and monolayer titanates. Special attention is devoted to their applications, ranging from energy storage solutions such as sodium and potassium ion batteries to environmental remediation efforts including ion-exchange processes. Moreover, we delve into the advantages and challenges of various synthesis routes, particularly emphasizing the trend toward non-fluorine-based precursors as a safer, more sustainable approach. A forward-looking discussion is included, highlighting the potential of these nanomaterials present in diverse scientific and industrial sectors. Future research directions aimed at optimizing these materials for electrochemical applications and potential integrations with other technologies are also considered.

## 2. Synthesis routes and mechanism

### 2.1. Exposed facets titania

Over the last fifty years, crystal facet engineering has been attracting increased attention as one of the most promising ways to enhance both the physical and chemical properties of solid-state materials. Exposing specific types of crystal facets of materials has been reported to be responsible not only for the increased catalytic activity, but also specialised optical and electronic properties.^20–23^ A similar approach has been applied to TiO_2_, considering its great potential in energy and environmental-related applications. Considerable effort has been made to develop a facile and straightforward synthetic protocol for the synthesis of TiO_2_ with specific control over particular crystal facets. Using both experimental and theoretical calculations, it is reported that several TiO_2_ physicochemical properties, such as catalytic activity, adsorption capability, surface atomic configuration, optoelectronic properties, and catalytic selectivity, could be affected by the type and degree of crystal facet exposure.^24–28^ Nevertheless, exposing the desired crystal facet of TiO_2_ during its crystal growth is a very challenging task. For instance, under equilibrium condition, most of the available anatase TiO_2_ crystals involve the thermodynamically stable {101} facet due to its low surface free energy (0.44 J m^−2^).^5,29,30^ High surface free energy facets, such as {001} (0.90 J m^−2^) and {010} (0.53 J m^−2^), quickly diminish during crystal growth due to their instability.^31^

In general, the controlled synthesis of titania with well-defined crystal facets can be achieved using different routes, *i.e.*, gas oxidation, epitaxial growth, spray-drying, topotactic transformation, crystallization transformation from amorphous TiO_2_ and wet-chemical syntheses such as hydrothermal, solvothermal or non-hydrolytic routes.^32–37^ However, hydrothermal and solvothermal synthetic routes are mostly preferred for the scalable fabrication of two-dimensional TiO_2_ nanostructures. This is primarily due to their ability to offer several beneficial advantages, such as low cost and strong ability to direct crystal growth and nucleation by only controlling reaction parameters. Typically, one of the most common strategies for exposing specific types of crystal facets in TiO_2_ is by the utilization of an appropriate capping agent during hydrothermal or solvothermal reaction.^38–41^ A capping agent is used to direct TiO_2_ crystal growth in a specific direction as the result of its preferential adsorption in a particular crystal plane. Other reaction parameters, such as the presence of Ti precursors, reaction time, temperature and the type of solvent, can influence the exposure of a particular crystal facet.^31^

The TiO_2_ {101} facet is one of the most common crystal facets in the anatase phase due to its low surface energy. Nevertheless, the truncated octahedral bipyramid with eight {101} facets and two {001} facets is found to be the most common crystal shape of anatase in the nature-based Wulff construction.^29,30^ Hence, many efforts have been made to develop synthetic routes for the formation of TiO_2_ that show only a {101} facet. One of the earliest approaches was to slow the reaction rate, which can be achieved by using Ti(iii) as the precursor rather than Ti(iv).^42–45^ In this approach, Ti(iii) is considered to be oxidized to Ti(iv) before it undergoes hydrolysis under hydrothermal conditions. Consequently, this would significantly slow the overall reaction rate due to the lack of dissolved oxygen. This approach was successfully applied by Hosono *et al.* when they prepared anatase TiO_2_ nanooctahedra with approximately 100% exposure of the {101} facets using TiCl_3_ as the precursor in the presence of sodium dodecyl sulfate (SDS) as a capping agent.^42^ Based on the result, it was also suggested that SO_4_^2−^ from SDS was responsible for the formation of an equilibrium crystal shape. This was proven by the formation of a slightly different slender pyramidal morphology when H_2_SO_4_ was used instead of SDS. In another report, a similar approach of utilizing TiCl_3_ as the Ti precursor was also reported in the hydrothermal synthesis of TiO_2_ with a {101} facet.^44^ In this approach, H_2_O_2_ was added as an oxidizing agent to produce the intermediate Ti(O_2_)_3_^2−^. In contrast, HCl was used to suppress the formation of the rutile TiO_2_ phase and to induce the crystal growth into the [101] direction. As a result, pyramidal anatase TiO_2_ with 100% exposure of {101} facets could easily be obtained.

**Fig. 2:**
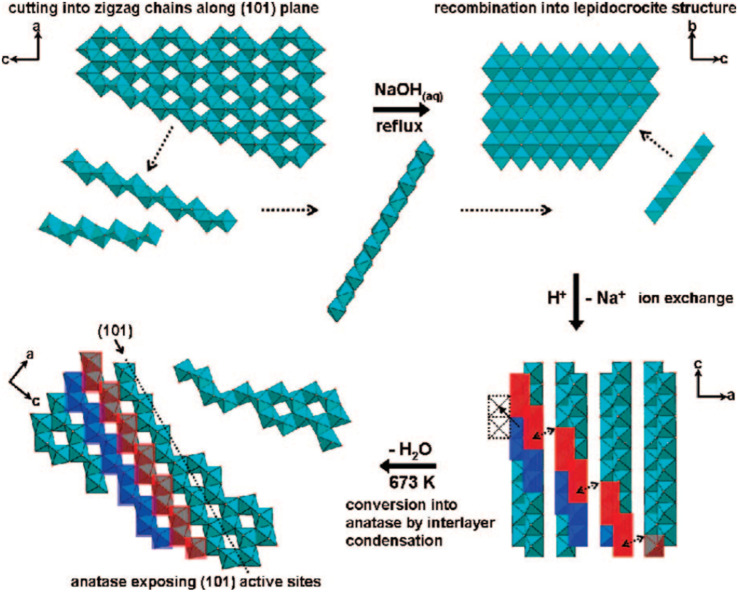
Schematic illustration of the conversion pathway of commercial bulk anatase to two-dimensional (101)-exposed anatase TiO_2_ nanosheet in the chimie-douce method. Reprinted with permission from ref. 48. Copyright 2008, American Chemical Society.

Furthermore, the highly exposed (101) facet of the TiO_2_ nanocrystals with octahedral morphology could also be obtained by transforming the amorphous one-dimensional TiO_2_ nanofiber *via* hydrothermal method at 160 °C.^46^ Based on the result, it is reported that such an approach was able to produce uniform octahedral TiO_2_ nanoparticles with high specific surface area (SSA) that predominantly exhibit the {101} facet and a small percentage of the {100} facet. Furthermore, Wu and co-workers have also successfully synthesized single-crystalline anatase TiO_2_ nanobelts with a high degree of surface exposure of the (101) facet by a hydrothermal transformation of the TiO_2_ powder in concentrated NaOH aqueous solution.^47^ It was found that the as-prepared (101)-exposed TiO_2_ nanobelts exhibited a lower rate of excitons recombination due to the significant enhancement in charge mobility, fewer localized recombination zones due to the reduction of unpassivated surface states, and improvement in the ability to trap photogenerated electrons. In another report, two-dimensional TiO_2_ with a high percentage of the {101} facet could also be obtained by converting both crystalline and amorphous TiO_2_*via* the chimie-douce (soft chemistry) method. For instance, Peng and co-workers have successfully converted commercial anatase TiO_2_ powder into a two-dimensional (101)-exposed anatase TiO_2_ nanosheet.^48^ Based on their results, it is believed that the bulk anatase crystals were able to be initially dissolved into several zigzag titanate chain building blocks in highly basic conditions, which could then be recrystallized back into the lepidocrocite structure where the exposure of the (101) surface is mostly preferred. Fig. 2 shows a schematic illustration for the conversion pathway of commercial bulk anatase to the two-dimensional (101)-exposed anatase TiO_2_ nanosheet.

Another approach that can be used to prepare two-dimensional TiO_2_ nanocrystals with high exposure of the {101} facets is by selecting the appropriate capping agent. For example, Yang and co-workers were able to develop a robust and straightforward synthetic protocol for TiO_2_ nanoleaves using a hydrothermal method at 140 °C, with titanium(iv) isopropoxide and triethylamine (Net_3_) as the Ti precursor and capping agent, respectively.^49^ Based on the result, the as-prepared TiO_2_ nanoleaves were able to be self-assembled into a facet-selective two-dimensional stacking structure along the [101] plane using Zn(ii)-porphyrin and the bidentate bipyridine. Recently, two-dimensional NTA was also successfully transformed into anatase TiO_2_ nanostructures with up to 95% exposure of the {101} facet using a solvothermal method with *tert*-butyl alcohol as the solvent.^50^ According to the report, it was found that the percentage of the {101} facet of the as-prepared TiO_2_ nanocrystals was proportional to its photocatalytic ability in hydrogen production. This superiority in catalytic performance was believed to be primarily due to the ability of the TiO_2_ {101} facet to serve as reduction sites with enriched electron populations.

**Fig. 3:**
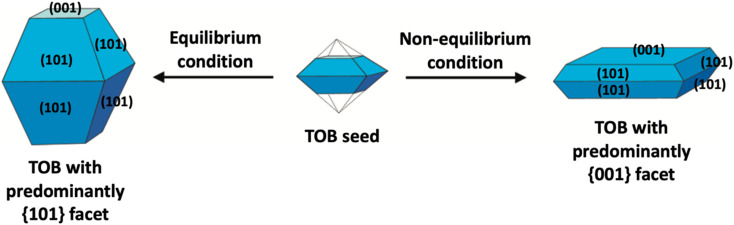
Schematic illustration of the TiO_2_ crystal evolution under equilibrium and non-equilibrium conditions.

In the literature, the fabrication of two-dimensional TiO_2_ nanostructures with a high exposure of {001} facets is by far the most exploited approach due to their high surface energy. In most cases, the synthesis of such material is carried out by preventing the crystal from growing in the [101] direction at the naturally occurring TOB shape according to Wulff construction. This can be achieved by making sure the crystal growth is carried out under the non-equilibrium condition at a kinetically controlled regime.^51,52^ In general, the TiO_2_ crystal nucleus would initially evolve as a TOB seed. Under equilibrium condition, TiO_2_ (ref. 33) facets would rapidly be diminished as the crystal prefers to grow into the thermodynamically stable TOB with predominately {101} facet. This is mainly because the {101} facet has significantly lower surface energy than the {001} facet. Under non-equilibrium conditions, the high surface energy {001} facets could be stabilized, resulting in the formation of a metastable two-dimensional TOB crystal with increased exposure of the {001} facets. Fig. 3 presents the schematic illustration for the TiO_2_ crystal evolution in both equilibrium and non-equilibrium conditions.

Traditionally, a non-equilibrium condition during TiO_2_ crystal growth could be kinetically achieved by controlling the temperature and ramping rate during the reaction. For example, Ahonen and co-workers were able to create a non-equilibrium condition by carrying out rapid heating and quenching of titanium(iv) isopropoxide *via* high-temperature (1200 °C) gas phase thermal oxidation.^53^ Based on this result, it was found that such a condition was able to form a well-faceted anatase TiO_2_ particle with the predominant exposure of the {001} facet. In another report, a similar rapid heating and quenching approach was also carried out using TiCl_4_ as the precursor.^54^ Here, the thermal oxidation process was done by liberating the Ti precursor vapor using argon bubbles and mixing with high-rate oxygen stream, where it was subsequently subjected to high temperature (1300 °C), which results in the formation of decahedral single-crystalline TiO_2_ particles with up to 40% exposure of the {001} facet. Both thermal oxidation temperature and its ramping rate were crucial in this synthetic method. A high exposure of the {001} facet could only be achieved when the annealing temperature was above 500 °C with a ramping rate above 16 °C min^−1^.^55,56^ This synthetic approach is also known to result in the formation of the rutile TiO_2_ phase as a byproduct.^53^ This method has been widely considered unsuitable for the scalable industrial production of such products.

**Fig. 4:**
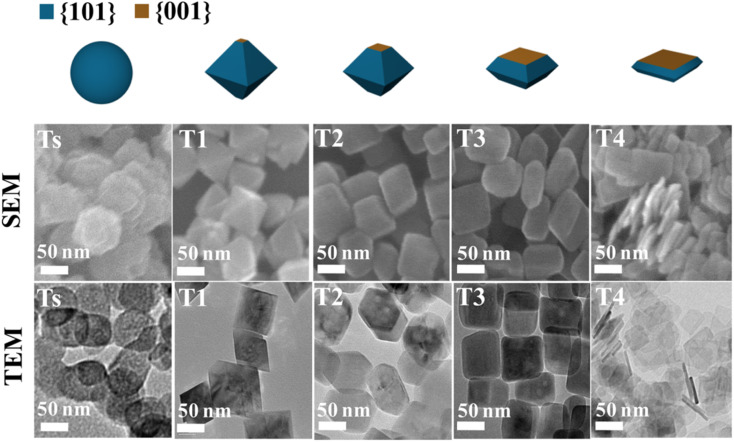
SEM and TEM images of the TiO_2_ nanocrystals with different degrees of the {001} facet synthesized at various ratios of HF/H_2_O. Reprinted with permission from ref. 63. Copyright 2017, American Chemical Society.

Many recent studies have considered the influence of various reaction dynamics for the synthesis of TiO_2_ crystals with a high exposure of the {001} facet in both aqueous and non-aqueous liquid phase systems. It has been shown that selecting a suitable titanium precursor, reaction temperature, pressure and solvent is important. The introduction of capping agents is essential to control the crystal nucleation.^21,28^ Among these factors, the type and amount of capping agent are considered as the most crucial contributing parameters in ensuring the high exposure of the {001} facet in TiO_2_. This is primarily due to the kinetics of crystal growth being exponentially proportional to the crystal surface energy.^51^ Typically, the specific surface energy of a crystal can be enhanced or reduced by selective adsorption of a capping agent on that particular crystal facet.^30^ As a result, the presence of a specific capping agent can significantly influence the final shape of the crystal. For the case of TiO_2_ with high exposure of the {001} facet, fluorine-based capping agents have been widely utilized due to their strong preferential interaction and ability to stabilize the {001} facet.^5^ During the past several years, different types of fluorine-based capping agents, such as HF, NH_4_F, NaF, and [bmim]-[BF_4_], have been effectively used to synthesize TiO_2_ with a high exposure of the {001} facet.^5,57–60^ Moreover, the utilization of fluorine-based Ti precursors, such as TiF_4_ and TiOF_4_, has also been reported to be able to produce TiO_2_ with high exposure of the{001} facet due to the simultaneous *in situ* generation of the F^−^ species.^61,62^ Furthermore, a study by Liu and co-workers revealed that the variation in the degree of co-exposure for both {101} and {001} facets could also be simply controlled by the ratio of HF/H_2_O during the solvothermal reaction.^63^ Based on the result, the percentage of {001} facet exposure was found to be proportional to the concentration of HF. Using this synthetic approach, two-dimensional TiO_2_ nanosheets with ≈92% exposure of the {001} facet were successfully fabricated and proven to exhibited exceptional ability as an antibacterial agent due to the presence of the {101}/{001} surface heterojunction. Fig. 4 shows the SEM and TEM images of the as-prepared TiO_2_ nanocrystals with different degrees of {001} facet exposure prepared at various HF/H_2_O ratios.

Additionally, a fluorine-free hydrothermal route with K-titanate nanowires and urea as the precursors was also introduced for the synthesis of two-dimensional TiO_2_ nanostructures with the {001} facet.^64^ In this synthetic approach, it was reported that the carbonate ions resulting from the decomposition of urea were found to be responsible for the formation of a high percentage of {001} facet (60%). In other reports, other inorganic species such as Cl^−^ and SO_4_^2−^ anions were also reported to be sufficient for directing the formation of TiO_2_ nanocrystals with a high exposure of the {001} facet.^65,66^ Recently, organic-based capping agents have also been explored for a similar application. For instance, Khalil and co-workers have also reported that an amine-based capping agent, *i.e.*, DETA, could also be utilized to expose the {001} facet during the hydrothermal synthesis of TiO_2_ with spindle-like morphology.^33,67^ In another study, Chen *et al.* successfully fabricated hierarchical sphere microstructures comprising the self-assembled two-dimensional ultrathin TiO_2_ nanosheet with nearly 100% exposure of the (001) facet using a mixture of isopropyl alcohol and DETA as the capping agent.^10^ Recently, a combination of HF and polymer-based capping agents, *i.e.*, poly(vinylpyrrolidone) (PVP), has also been reported to be utilized for the synthesis of TiO_2_ nanomosaics comprising two-dimensional TiO_2_ with a high percentage of exposure for {001} facet.^68^ In this report, it was believed that the large and bulky polymeric PVP molecules could serve not only as the linker between TiO_2_ nanosheets, but also prevent them from stacking together along the *c*-axis.

### 2.2. Monolayer titanate

Since 1998, Sasaki *et al.*^17^ have studied the single layered nanosheets prepared by chemical exfoliation of lepidocrocite-like titanate, in which the solid-state reaction was the main method used to synthesize the parent compound at that time.^69^

Since the reaction occurs in the solid state, a high temperature process (800–1500 °C) is usually required to induce the reaction of solid precursors.^70^ To ensure a uniform reaction, crushing and grinding are usually performed with a mortar and pestle to produce a thorough mixture of precursors, while ball milling could be used for a larger quantity. To help with the homogenisation, a small amount of solvent such as alcohol or acetone can be added, in which it will evaporate after the precursors are perfectly mixed.^70^ Instead of using additional solvent, pelleting can be performed as an alternative to produce a good contact between the precursors. The rate of the solid-state reaction can be controlled by adjusting the temperature and by considering certain properties of the precursor, such as the surface area, its reactivity, and morphology. To increase the reactivity, a molten salt is often used as an additive and solvent.^71^

**Fig. 5:**
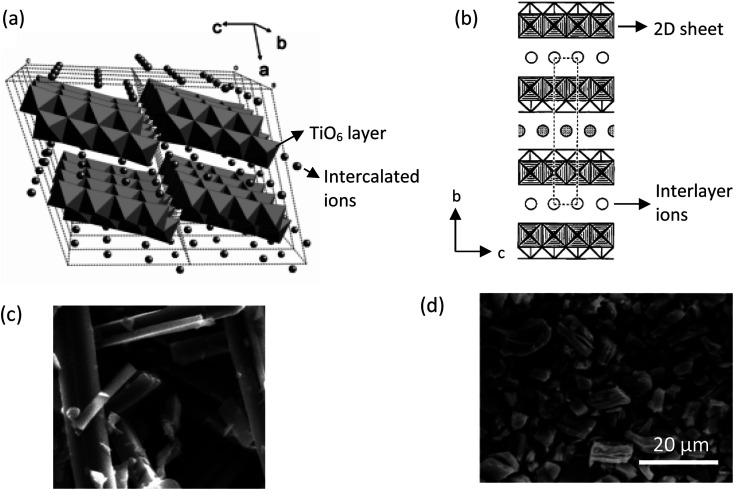
(a) Crystal structure of the fibrous-like titanate (monoclinic);^72^ (b) crystal structure of the lepidocrocite-like titanate (orthorhombic) viewed along the *a*-axis;^73^ (c) FESEM image of fibrous-like titanate;^73^ (d) FESEM image of lepidocrocite-like titanate.^74^ Panel (a) adapted with permission.^72^ Copyright © 2010, American Chemical Society. Panel (b and c) adapted with permission.^73^ Copyright © 1995, American Chemical Society. Panel (d) adapted with permission.^74^ Copyright © 1998, American Chemical Society.

The common solid-state reaction of titania and alkali salt precursors, such as CsNO_3_, Cs_2_CO_3_, and K_2_CO_3_ often results in a fibrous (monoclinic) titanate structure. In 1987, Grey *et al.*^69^ discovered a new type of titanate compound using a non-stoichiometric reaction, where the resulting product has a layered structure of the lepidocrocite-like (orthorhombic) titanates. Fig. 5 shows the crystal structures and scanning electron microscopy (SEM) images of the fibrous and lepidocrocite-like titanate compounds.

In the Grey *et al.* method, a TiO_2_ : CsNO_3_ molar ratio of around 1 : 2.8–3.2 was mixed, followed by heating at 800–1050 °C for 0.5–20 hours, producing a white powder of lepidocrocite-like caesium titanate with the chemical formula of Cs_*x*_Ti_2−*x*/4_□_*x*/4_O_4_, where *x* is about 0.61–0.65 and □ represents a titanium vacancy. The procedure has been further developed by Sasaki *et al.*, who used Cs_2_CO_3_ and TiO_2_ with a molar ratio of 1 : 5.3 exhibiting lepidocrocite-like lamellar sheets.^73^ Besides the caesium-based precursor, Sasaki *et al.* also utilised Li_2_CO_3_ and K_2_CO_3_ in place of Cs_2_CO_3_ for the reaction with titania powder.^75^ The reaction of Li–K-based titanates can be enhanced by K_2_MoO_4_ molten flux process, which acts as an excellent heat transfer medium. The slow-cooling procedure in the flux process yields very large nanosheets of up to 30 microns, while the solid-state reaction typically produces nanosheets of ≈0.5–1 microns.^76,77^

The layered lepidocrocite-like titanate needs to be exfoliated to produce monolayer nanosheets. Sasaki *et al.* showed a facile two-step ion-exchange method to exfoliate the nanosheets. Firstly, the interlayer caesium or potassium ions were etched with acid and replaced by H^+^ ions. For complete removal of alkali ions, a repeated acid treatment with a fresh solution was required in which 98% of the alkali ions were removed after three daily cycles.^72^ As a result, lepidocrocite-like titanate with a high cation-exchange capacity was produced after the acid ion-exchange reaction, exhibiting a similar smectite clay-like behaviour.

**Fig. 6:**
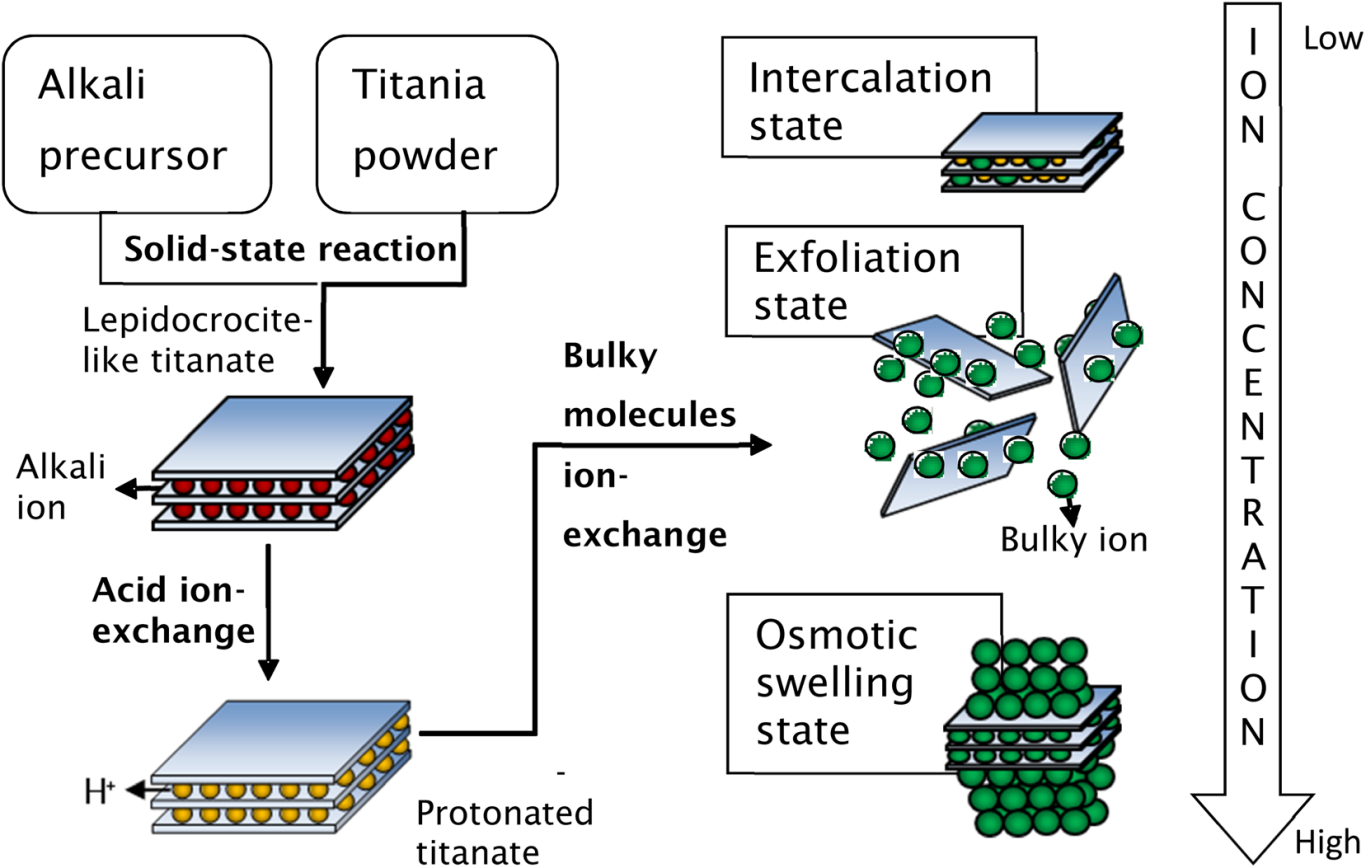
Schematic representation of the synthesis of single-layer titania nanosheets *via* chemical exfoliation process.^80^ Copyright © 2017 IOP Publishing.

Secondly, the exchange of bulky ions with TBA^+^ or TMA^+^ ions was conducted to assist the complete exfoliation of protonated layered titanate. The properties of the resulting compounds were similar to those of smectite clays such as montmorillonite, hectorite, and saponite, in which the basal spacing could be expanded (swollen) by the intercalation of guest molecules. Depending on the concentration of bulky ions, the titania nanosheets can be in intercalated, exfoliated, or osmotic-swelling states,^17^ as shown in Fig. 6. An extensive study on the exfoliation of nanosheets has been conducted by Sasaki *et al.*^17^ By controlling the molar ratio of TBA^+^ to H^+^, the state of the titania nanosheets can be adjusted from intercalation → exfoliation → swelling. For caesium-based titania nanosheets, the intercalation state occurs when the ratio of TBA^+^/H^+^ is less than 0.5, as examined by SAXS. The interlayer spacing of nanosheets increases as the number of bulky ions increases, leading to infinite interlayer spacing and the induction of exfoliation. The fully exfoliated state occurs within the TBA^+^/H^+^ ratio of 1–5. When the ratio of TBA^+^/H^+^ exceeds 5, a multilayer arrangement of lamellar sheets occurs, exhibiting a diffuse double layer through osmotic swelling. During the osmotic-swelling state, the interlayer spacing becomes smaller, leading to sheet coagulation as the number of ions increases. One must note that the ratio of TBA^+^/H^+^ varies for each type of nanosheets depending on the stoichiometry and charge density of the layered compounds.^75,78,79^ This chemical exfoliation method may produce very large nanosheets if gentle stirring or shaking is applied during the exfoliation process.^75^

Besides the chemical exfoliation method, the exfoliation can be conducted by mechanical approaches such as supercritical fluid exfoliation^81^ and ultrasonication assisted ion-exchange.^82^ High energy jets created by the implosion of bubbles during ultrasonication break up the layered nanosheets in a relatively short time, although it also reduces the lateral size of the nanosheets. Meanwhile, the supercritical fluid method utilises the fluid expansion to exfoliate the nanosheets. A supercritical fluid is any compound at a temperature and pressure above its critical point, where the intermediate phase (which can effuse through solids like a gas and dissolve materials like a liquid) occurs. At the beginning, the layered nanosheets are intercalated by the supercritical fluid. In this state, the exfoliation can easily occur by applying thermal stress to the intercalated nanosheets. However, the exfoliated nanosheets may be restacked upon cooling down; hence, a faster cooling rate is preferable. The highest yield of exfoliated nanosheets by this method, however, was estimated to be only 10%.^81^

While the chemical exfoliation method uses a top-down approach from precursors synthesized by a solid-state reaction, other researchers synthesised single-layered nanosheets using bottom-up approaches such as the electron beam deposition (EBD) of titania and oxygen atoms under ultra-high vacuum^83^ and sol–gel method.^18,84^ Ti was deposited by e-beam deposition on (1 × 2)-Pt(110) at room temperature (*p*O_2_ = 1 × 10^−4^ Pa), followed by post-annealing treatment at 700 K and cooling down in oxygen (*p*O_2_ = 1 × 10^−4^ Pa), resulting in titania nanosheets with 3.9 × 1.6 nm lateral size.^83^ A sol–gel solution of titania nanosheets can be synthesised by reacting the titanium precursor (*i.e.*, TiF_4_ and (NH_4_)_2_[TiO(C_2_O_4_)_2_]) with an aqueous solution of KOH or NaOH. The resulting product was a small multi-layered nanosheet; hence, a bulky molecule such as TBAOH or TMAOH was still required to exfoliate the nanosheets.^85,86^ On the contrary, a sol–gel synthesis of TIP with a large excess of aqueous bulky molecule solution of TMAOH exhibited a high yield of diamond or rhombic-shaped monolayered nanosheets.^18^ The bulky molecule served as the reactant for the acid–base reaction with titanic acid, as well as providing enough ionic charge to maintain the exfoliation of nanosheets. Compared to the chemically exfoliated nanosheets, the sol–gel synthesis usually produces relatively small nanosheets of less than 50 nm in lateral size.

**Fig. 7:**
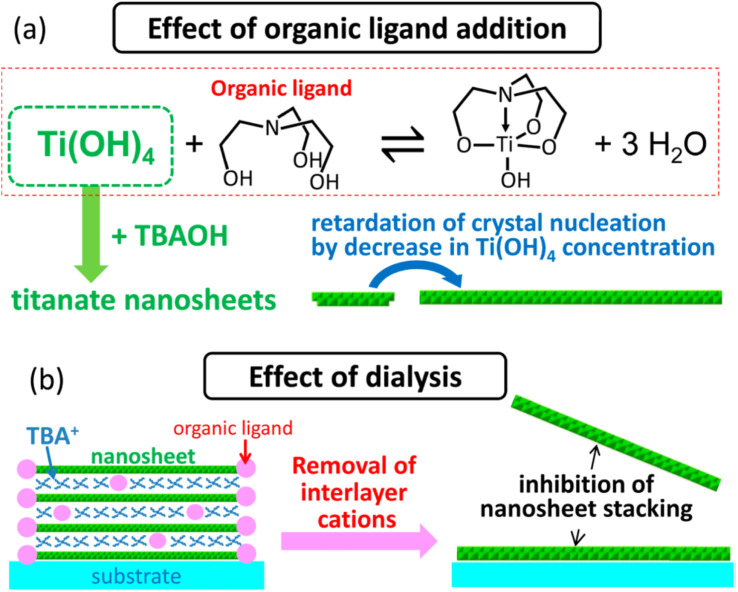
The illustration of (a) the effect of the organic ligand in the crystallisation of the titania nanosheets and (b) inhibition of nanosheets restacking by dialysis.^87^ Copyright © 2015, American Chemical Society.

Ban *et al.*^87^ further developed the sol–gel synthesis by using an organic ligand (*e.g.*, triethanolamine and lactic acid) to form a titanium complex, hence retarding the nucleation of titania nanosheets while promoting growth in the lateral direction. This method created ≈100 nm diamond-shaped titania nanosheets after several days of reaction in the autoclave. However, the organic ligand may also cause the restacking of nanosheets during evaporation; hence, it should be removed by dialysis. Ban's sol–gel synthesis of large nanosheets is illustrated in Fig. 7.

**Fig. 8:**
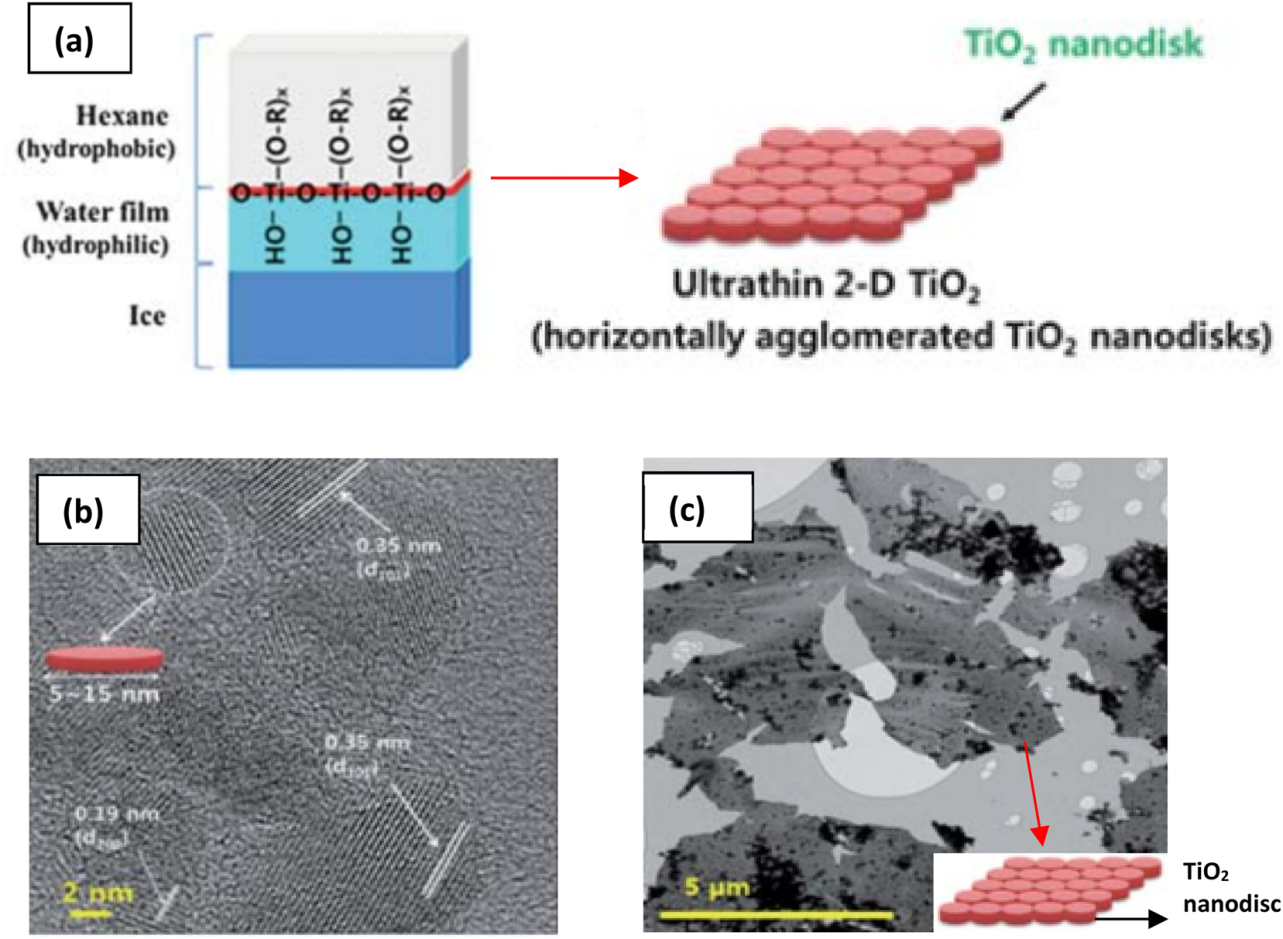
(a) Schematic illustration of the 2-D TiO_2_ formation on the hexane/ice interface.^84^ TEM images of a nanosheet consisting of horizontally agglomerated TiO_2_ nanodiscs:^84^ (b) HR-TEM image of nanodiscs, (c) TEM image of nanosheets. Reproduced from ref. 84 with permission from the Royal Society of Chemistry.

**Fig. 9:**
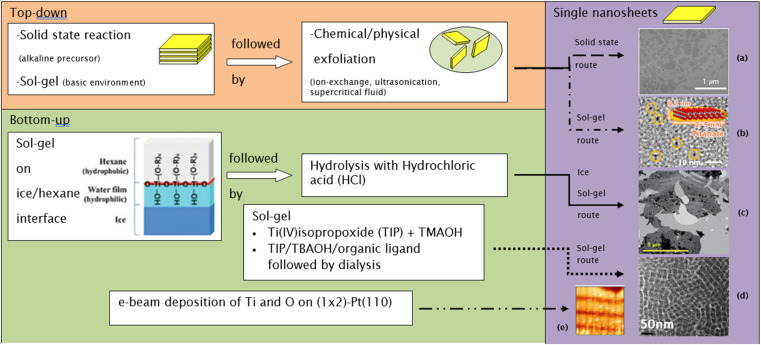
Several routes to synthesize single-layer titania nanosheets: (a) TEM image of nanosheets made by the solid-state route,^14^ (b) bright-field TEM image of nanosheets made by the sol–gel route,^86^ (c) TEM image of nanosheets made by the ice sol–gel route,^84^ (d) TEM image of nanosheets made by the sol–gel route,^18^ (e) high-resolution STM image of nanosheets made by the e-beam deposition route (13.6 nm × 13.6 nm; bias voltage = 0.42 V; *I*_T_ = 0.9 nA).^83^ Panel (a) adapted with permission.^14^ Copyright © 2010 WILEY-VCH Verlag GmbH & Co. KGaA, Weinheim. Panel (b) adapted with permission.^86^ Copyright © 2013, American Chemical Society. Panel (c) reproduced from ref. 84 with permission from the Royal Society of Chemistry. Panel (d) adapted with permission.^83^ Copyright © 2006 by the American Physical Society.

The need to confine the growth of titanate in the lateral dimension has been developed by another group. Sol–gel synthesis at the hydrophobic/hydrophilic (*i.e.*, hexane/ice) interface can be deployed to create large nanosheets, as illustrated in Fig. 8.^84^ These nanosheets contain several small nanodiscs with ≈5 to 15 nm in lateral size, which are agglomerated horizontally. The single-layered nanosheets structure was confirmed by atomic force microscopy (AFM), whereas the nanosheets are only ≈0.5–1 nm in thickness. After hydrolysis by HCl, the anatase TiO_2_ structure was formed, as characterised by X-ray diffraction (XRD). A schematic of the synthesis route to TiNS is outlined in Fig. 9 and it is summarized in Table 1.

Summary of the synthesis methods for single-layered titania nanosheets

**Table:** 

Top-down approach				
Synthesis of layered nanosheets	Exfoliation method and its additive	Chemical formula	Lateral size	Ref.
**Solid-state reaction**				
Cs_2_CO_3_ + TiO_2_ → Cs_*x*_Ti_2−*x*/4_□_*x*/4_O_4_ (*x* ≈ 0.7; □ = titanium vacancy)	Ion-exchange at 25 °C for 2 weeks by 0.00825 to 0.0825 mol L^−1^ aqueous solution of (tetrabutylammonium hydroxide) TBAOH	Ti_0.91_O_2_^0.36−^	≈0.1–1 μm	17
Reaction at 800 °C for 20 h (2 times)				
Cs_2_CO_3_ + TiO_2_ + MgO → Cs_*x*_Ti_2−*x*/2_Mg_*x*/2_O_4_ (*x* ≈ 0.7)	Ion-exchange at 50 °C for 1 week by 5 wt% aqueous solution of TBAOH or (tetramethylammonium hydroxide) TMAOH	Ti_0.825_O_1.825_^0.35−^	≈0.1–1 μm	88
Reaction at 800 °C for 1 h followed by 2 times heating at 950 °C for 20 h				
K_2_CO_3_ + TiO_2_ + Li_2_CO_3_ → K_*x*_Ti_2−*x*/3_Li_*x*/3_O_4_ (*x* ≈ 0.8); (with K_2_MoO_4_ as flux melt)	Ion-exchange at 25 °C for 2 weeks by 0.0125 to 0.025 mol L^−1^ aqueous solution of TBAOH or TMAOH	Ti_0.87_O_2_^0.52−^	0.5–2 μm; average ≈ 1 μm for TBAOH and 10–30 μm for TMAOH	75
Reaction at 1200 °C for 10 h, followed by slow cooling (4 °C h^−1^) until it reaches 950 °C				
Na_2_CO_3_ + TiO_2_ → Na_2_Ti_3_O_7_	Ion-exchange by methylamine at 60 °C for 6 d, followed by propylamine at 60 °C for 6 d	Ti_3_O_7_^2−^	≈0.1–1 μm (rectangular)	89
Reaction at 900 °C for 24 h				
Cs_2_CO_3_ + TiO_2_ → Cs_*x*_Ti_2−*x*/4_□_*x*/4_O_4_ (*x* ≈ 0.7; □ = titanium vacancy); reaction at 800 °C for 20 h (2 times)	Ion-exchange by TBA^+^ ion assisted with ultrasonication (60–300 W, 2–30 min)	Ti_0.91_O_2_^0.36−^	≈0.1–0.2 μm	82
K_2_CO_3_ + TiO_2_ + Li_2_CO_3_ → K_*x*_Ti_2−*x*/3_Li_*x*/3_O_4_ (*x* ≈ 0.8) (with K_2_MoO_4_ as flux melt). Reaction at 927 °C for 10 h (spontaneous cooling)	Supercritical DMF exfoliation (400 °C, 15 min)	Ti_0.87_O_2_^0.52−^	≈5–20 μm	81

**Sol–gel followed by ion exchange**				
(NH_4_)_2_[TiO(C_2_O_4_)_2_] + KOH → K_1.1_H_0.9_Ti_2_O_5_·2.6H_2_O (1 day, 22–80 °C)	Ion-exchange by aqueous solution of TBAOH at 22 °C	Ti_2_O_5_^2−^	≈10–20 nm	85
TiF_4_ + NaOH → Na_0.8_Ti_1.8_□_0.2_O_4_·*y*H_2_O (*y* < 1.17) (3 days, 22 °C)	Ion-exchange by aqueous solution of TBAOH at 22 °C	Not available	≈2–5 nm	86

**Table:** 

Bottom-up approach			
Method	Chemical formula	Lateral size	Ref.
Reflux of Ti(iv)isopropoxide (TIP) + aqueous solution of tetramethylammonium hydroxide (TMAOH); (5 min to 24 h, 100 °C)	(TMA)_*x*_Ti_2−*x*/4_□_*x*/4_O_4_ (*x* ≈ 0.7)	Diamond shape with a diagonal length of (27.3, 19.1) nm to (7.7, 5.5) nm	18
TIP + organic ligand (*e.g.*, triethanolamine or lactic acid) + tetrabutylammonium hydroxide (TBAOH) heated in autoclave at 80 °C for 1–7 days, followed by dialysis with water for 2 days	(TBA, H)_0.7_Ti_1.825_O_4_·*x*H_2_O	Diamond shape with ≈100 nm lateral size	87
Sol–gel of hexane + TIP + ice granule interface, followed by hydrolysis with HCl	TiO_2_	≈5 μm consist of 5–15 nm nanodiscs	84
e-beam deposition on (1 × 2)-Pt(110); Ti was deposited at room temperature (*p*O_2_ = 1 × 10^−4^ Pa), followed by post-annealing treatment at 700 K and cooling down in oxygen (*p*O_2_ = 1 × 10^−4^ Pa)	TiO_2_	3.9 × 1.6 nm	83

## 3. Properties

### 3.1. Physical properties

#### 3.1.1. Exposed facets titania

**Fig. 10:**
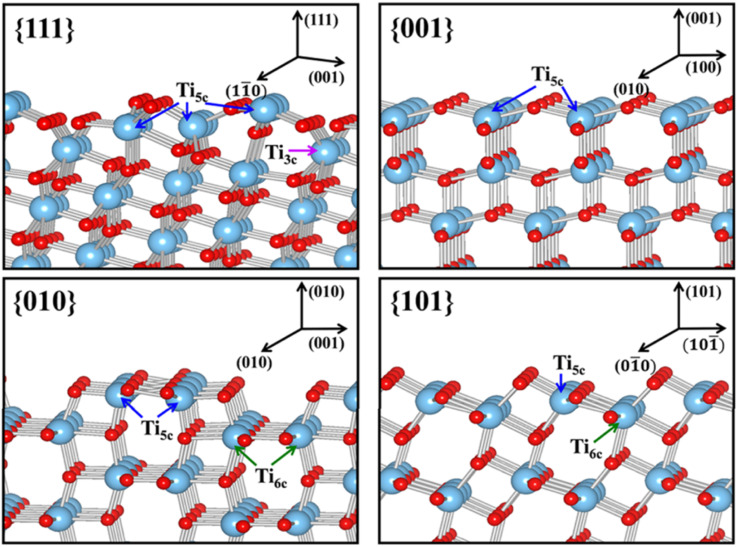
Slab model for the surface structures of the relaxed stoichiometric TiO_2_'s {111}, {001}, {010} and {101} facets. Reprinted with permission from ref. 100. Copyright ©2013, American Chemical Society.

Both optical and electronic properties have been widely considered as the most common direct consequences for the exposure of a specific crystal facet in 2D TiO_2_ nanostructures. Typically, this can easily be observed by the alteration of both bandgap and band edge location. According to recent studies, the exposure of the TiO_2_ {001} facet while diminishing the existence of the {101} facet may contribute to the reduction of the TiO_2_ optical band gap.^44,90,91^ For instance, Liu *et al.* calculated that the optical bandgap of TiO_2_ nanostructures with 5%, 60%, and 92% exposure of the {001} facet was found to be 3.33, 3.29, and 3.16 eV, respectively.^92^ A similar bandgap narrowing due to the exposure of the {001} facet was also observed elsewhere.^93,94^ According to theoretical calculation using DFT, such reduction was most likely because the {101} facet possesses a slightly higher CB than the {001} facet.^95–97^ Such a phenomenon might also have occurred due to the presence of oxygen vacancies as a result of the unique surface atomic arrangement.^98,99^ Furthermore, a similar band structure alteration was also observed in other high-index facets. For example, using both experimental and theoretical estimations, Xu and co-workers revealed that TiO_2_ with the {111} facet exhibited a higher conduction band minimum in comparison to TiO_2_ with {001}, {101}, and {010} facets.^100^ It is believed that such a phenomenon was partially attributed to the large percentage of undercoordinated Ti and O atoms at the surface of the {111} facet. Fig. 10 presents the slab model for the surface structure of TiO_2_ at different crystal facets.

Furthermore, the unique arrangement of atoms at the surface of TiO_2_ from the exposure of different facets may also influence the efficiency of the charge carrier separation. Traditionally, the prevention of the fast photogenerated electron–hole recombination of TiO_2_ was typically done by heterojunction or the application of sacrificing agents.^101,102^ However, recent studies have suggested that crystal facet engineering of TiO_2_ could also be used as an effective strategy to avoid such issue.^103–106^ For example, the high surface energy {001} facet has been proven to exhibit superior ability in ensuring efficient separation of photoexcited charge carriers. It is believed that such a phenomenon was partly due to the presence of surface defects, *e.g.*, oxygen vacancies, which could efficiently mediate the interfacial electron transfer.^21^ Additionally, a high density of undercoordinated Ti atoms and large Ti–O–Ti bond angles at the surface of the {001} facet has also been considered as one of the main contributors for such phenomenon.^5^ Recently, the contribution of two or more co-existing facets has also been associated with a more efficient charge separation. For instance, Yu *et al.* reported that the co-exposed {001} and {101} facets were found to exhibit a synergistic effect that was responsible for the enhancement of the photocatalytic activity of the 2D TiO_2_ nanosheet.^107^ Using DFT calculation, it was revealed that the enhancement in the photoactivity was primarily due to the formation of a surface heterojunction between the {001} and {101} facets as a result of their band alignment. This is possible since the positions of CBM and VBM of the {001} facet were found to be more positive than that of the {101} facet.^92^ As a result, the photogenerated electron tends to be thermodynamically transferred to {101}, while the hole preferentially moves in the opposite direction.

Another physical characteristic that may be influenced by the exposure of certain facets in 2D TiO_2_ nanostructures is their capability in substrate adsorption. It is reported that the specific geometric structure and atomic arrangement at the surface of a particular TiO_2_ facet could affect the interaction between TiO_2_ and various types of substrates, *e.g.*, water, methanol, CO_2_, or other small molecules.^108–110^ One of the widely accepted rationalizations for such a phenomenon was the fact that certain facets exhibit different degrees of oxygen vacancy and undersaturated Ti coordination. For example, the surface of TiO_2_'s {001} facet is widely known to have 100% undercoordinated Ti-5c atoms and half saturated Ti-6c atoms. In contrast, the {101} facet exhibits half of the undercoordinated Ti-5c atoms and half saturated Ti-6c atoms.^31^ The {001} facet is also reported to have a larger stoichiometric amount of the surface hydrophilic Ti^3+^ and surface OH groups than the {101} facet.^111^ As a result, the sorption capacity of the {001} facet for water or other polar molecules is expected to be higher than that of the {101} facet. A similar superiority in the sorption capacity of the {001} facet over different facets was also observed elsewhere for the absorption of Cr_2_O_7_^2−^ and arsenic.^112,113^ It is also worth noting that the specific surface area and particle size may both contribute to the overall sorption capacity.

#### 3.1.2. Monolayer titanate

**Fig. 11:**
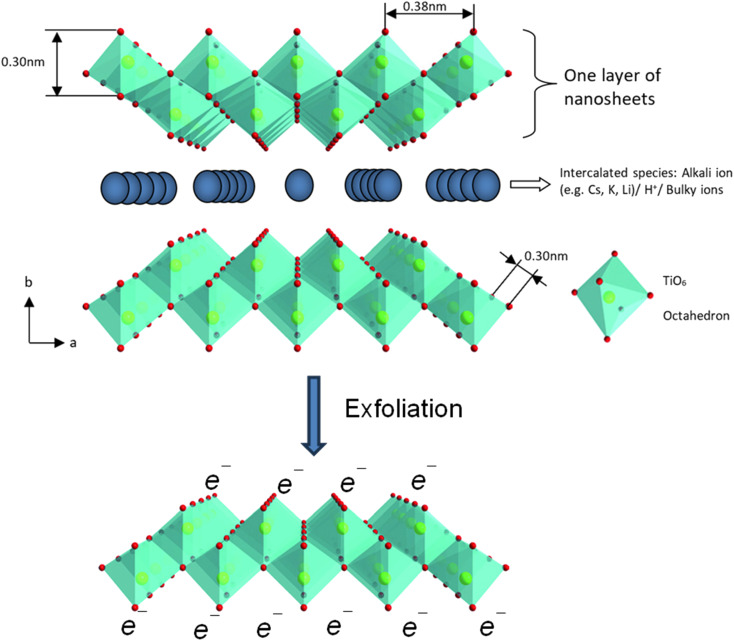
The polyhedral representation of the crystal structure of layered lepidocrocite-like titania nanosheets viewed down along the *c*-axis and mono-layer titania nanosheets after exfoliation. Adapted from ref. 88 with permission from the Royal Society of Chemistry.

The properties of chemically exfoliated titania nanosheets are related to its chemical formula, in which the precursors (*i.e.*, lepidocrocite-like titanate) have a general formula of Cs_*x*_Ti_2−*x*/4_□_*x*/4_O_4_, where *x* is around 0.7 and □ is titanium vacancy^114,115^ for the caesium-based and A_*x*_Ti_2−*x*/3_Li_*x*/3_O_4_ nanosheets, where *x* ≈ 0.8 for A = K (potassium) and *x* ≈ 0.75 for A = Rb.^17^ The detailed crystal structures of these compounds are shown in Fig. 11. After alkali ion removal, the nanosheets are negatively charged with the general formula of Ti_0.91_O_2_^0.36−^ for nanosheets derived from Cs_*x*_Ti_2−*x*/4_□_*x*/4_O_4_.

**Fig. 12:**
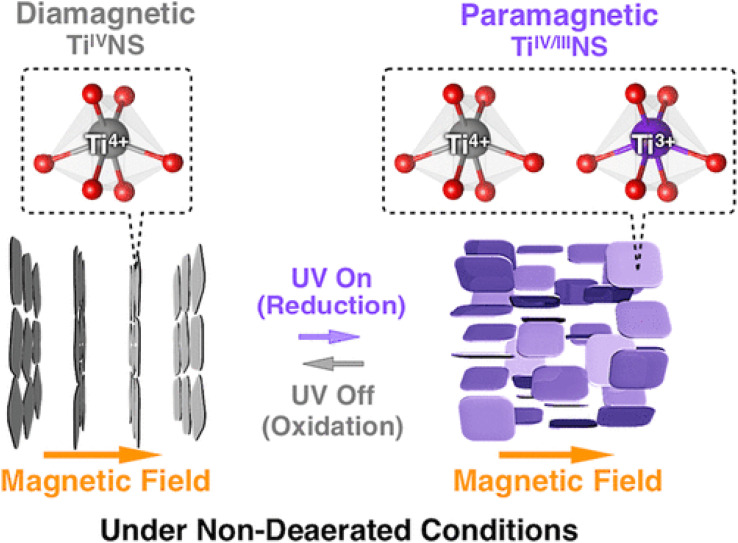
The orthogonal and parallel magnetic orientation switching of titania nanosheets *via* photoreduction and oxidation.^118^ Copyright © 2018, American Chemical Society.

The absorption peak wavelength of the Ti_0.91_O_2_^0.36−^ nanosheets is blue-shifted to around 265 nm, as compared to anatase TiO_2_ at 377 nm.^8^ It is known that the molar absorption coefficient or molar extinction coefficient (*ε*) is 2.2 × 104 mol^−1^ dm^3^ cm^−1^ at 265 nm.^116^ This blue shift also occurs in sol–gel diamond-like titania nanosheets, which has the peak around 250 nm.^18^ It was concluded by both researchers that the quantum confinement significantly contributes to the optical properties of titania nanosheets especially due to the transition from the 3D to 2D structure. Using spectroscopic ellipsometry, the refractive index of the Ti_0.87_O_2_^0.52−^ nanosheets was found to be around 2.1 at 600 nm and the extinction coefficient of a thin film (*k*) was nearly zero.^117^ The titanate nanosheets in the structure possess diamagnetic properties, which may have aligned itself in the 2D plane perpendicular to the magnetic flux direction due to the highly anisotropic magnetic susceptibility.^118^ The magnetic susceptibility can be altered *via* UV photoreduction of Ti^IV^ to Ti^IV/III^ nanosheets, which exhibits paramagnetic properties. It changes the orientation from the orthogonal to parallel direction when exposed to the magnetic field, as depicted in Fig. 12.

The electronic band gap energy of Ti_0.91_O_2_^0.36−^ nanosheets was 3.84 eV, as estimated by *in situ* UV-vis spectroscopy,^119^ which is 0.6 eV larger than that of anatase titania.^120^ Compared to anatase TiO_2_, TiNS has a slightly higher conduction band at −1.27 eV *vs.* Ag/Ag^+^ and significantly lower valence band at 2.53 eV *vs.* Ag/Ag^+^.^119^ The exfoliated titania nanosheets has a larger band gap than its lepidocrocite-like titanate precursor, hence further confirming the effect of quantum confinement. Compared to the anatase TiO_2_, stronger UV light is required to activate the photocatalytic capability of titania nanosheets. To overcome the large band gap, metal and non-metal doping are often introduced for narrowing the band gap. Due to a titanium vacancy in the structure, co-doping is possible for titania nanosheets.^119^ One cation is used to replace the interlayer ions, while the other may co-substitute Ti^4+^ in octahedral sites. Fan *et al.*^121^ utilised the photocatalytic properties of titanate titania nanosheets by doping with platinum nanoparticles *via* photoreduction of Pt(IV) ions, which is indicated by the colour changing from white to dark grey. Besides the precious metal, titania nanosheets have been doped by Fe, Ni, Co, Nb, and Mn ions for metal doping and nitrogen ions for non-metal doping.^119^ Few studies have examined non-metal doping. Thus, an exploration of co-doping by a non-metal dopant should be conducted for further research.

In terms of thermal stability, the monolayered titania nanosheets maintained its structure up to 800 °C before it transformed to anatase TiO_2_.^119^ The stability was reduced with increasing number of stacks of the titanate layers, in which 10 stacks of nanosheets transformed into anatase TiO_2_ at around 400 °C. The 2D structure limits the diffusion of atoms, hampering the 3D formation of the anatase structure. Meanwhile, the electrical conductivity depends on the relative humidity, where it increases by about 5 orders of magnitude from 45% to 95% relative humidity.^122^ Water molecules adsorbed on the titanate surface can bridge the electrical transport in the lateral dimension.

### 3.2. Chemical properties

#### 3.2.1. Exposed facets of titania

The unique geometric structure and atomic arrangement at the surface of 2D TiO_2_ nanostructures with certain exposed facets have also been associated with the enhancement of their catalytic reactivity. Recently, the ability to control the surface and electronic properties *via* crystal facet engineering of 2D TiO_2_ nanostructures has attracted much attention as a way to improve their performance in various applications, especially in catalysis or light-harvesting devices. It is believed that the presence of undercoordinated Ti atoms and the number of oxygen vacancies at certain crystal facets has a significant influence in dictating both the kinetics and thermodynamics of the reaction. For instance, TiO_2_ with a high exposure of the {001} facet has been well-documented to be more reactive towards water dissociation and more effective for facilitating photoredox reactions than that with a high exposure of the {101} facet.^30,110^ In another report, Amano *et al.* have also reported that the performance of 2D decahedral single-crystalline TiO_2_ with a high exposure of the {001} facet in hydrogen evolution *via* the water splitting reaction was superior to that of the commercial P25 Degussa TiO_2_ powder.^54^ Recently, Khalil and co-workers have also proven that the exposure of the {101} facets was responsible for the enhancement in the photocatalytic activity of the nano Au–TiO_2_ heterostructures for the photodegradation of organic pollutants.^33^ Based on this result, the synergistic effect between the surface plasmon resonance phenomenon and the exposure of the {001} feature was able to significantly increase the reaction rate by ten-folds. Furthermore, they also reported that a similar enhancement in photocatalytic activity by the co-exposure of the TiO_2_ (101) and (001) facets was also observed in the photocatalytic reduction of bicarbonate using CdSe–TiO_2_ nanostructures.^67^

In addition to the enhancement in the catalytic activity, the exposure of certain crystal facets in 2D TiO_2_ nanocrystals was also reported to be responsible in the variation of the catalytic selectivity. For instance, the selectivity of the toluene conversion to benzaldehyde can be enhanced by simply increasing the percentage of exposure for the {001} facet in the two-dimensional TiO_2_ nanosheet.^62^ According to the report, the selectivity for the formation of benzaldehyde could be increased by up to 93% (yield of 26%) by exposing 50% of the {001} facet. In another report, Liu and co-workers reported that the exposure of the {001} facet could also influence the selective adsorption and photocatalytic activity towards azo dyes.^123^ It was revealed that TiO_2_ with a low exposure of the {001} facet (P25 titania, 5% of exposed {001} facet) showed a preferential photocatalytic decomposition of MO. Meanwhile, TiO_2_ with a high exposure of the {001} facet favors the degradation of MB. In the literature, this selectivity was believed to originate from the unique surface atomic configuration of the {001} facet, which results in the alteration of surface characteristics such as the surface charge, Lewis and Brønsted acidity, and exposed functional groups.^124,125^ It is suggested that the spatial distribution of the redox sites due to the preferential separation of photogenerated charge carriers at certain crystal facets may also contribute to the aforementioned catalytic selectivity.^126,127^

#### 3.2.2. Monolayer titanate

The high reactivity of interlayer alkali metal ions such as Cs^+^ and K^+^ is advantageous for the ion exchange reaction with protons that facilitate the exfoliation of titania nanosheets. The cation exchange capability of chemically exfoliated titania nanosheets is beneficial in energy storage applications; for example, it can be used for lithiation and de-lithiation in a lithium-ion battery.

In terms of colloidal stability, a net negative charged on the titanate surface is formed after the removal of alkali metal ions, in which it is stable in basic solution with the point of zero charge at pH 8 and zeta potential of −37 mV at pH 10–13.^128^ In TBAOH or TMAOH solutions, the colloidal suspension of chemically exfoliated titania nanosheets is stable for more than 6 months. It was observed that sol–gel titania nanosheets are more stable due to the smaller particle size. A stable colloidal suspension is convenient for the deposition process, in which the controlled deposition of titania nanosheets can be realised by Langmuir–Blodgett procedure and electrostatic layer-by-layer assembly.^129^ Alternatively, an amount of titanate can be drop-casted on the surface, yielding a film with cation-conducting properties.^130^ Electrophoretic deposition can also be performed to decorate the electrode *via* the negative surface charge of chemically exfoliated titania nanosheets.^131^ The negative surface charge is also exhibited in sol–gel titania nanosheets.^132^ When an electrophoretic deposition technique combines with mechanical stimulation, small sol–gel titania nanosheets can be inserted within titanate nanotubes to create a hierarchical structure^132^ of titania nanosheets.

Modification of the surface functional group of titania nanosheets has been studied.^128^ In an aqueous solution, chemisorbed and physiosorbed water molecules are attached to the surface of titanate, leading to a hydroxylated surface, where the functionalisation can be performed *via* these hydroxyl group titania nanosheets. Generally, the modification of the hydroxyl group of titanate can be approached *via* hydrolysis with silane groups, esterification with carboxylic acid, peroxo-titanium complex formation by H_2_O_2_, acid–base reaction, and formation of admicelles by surfactant.^133,134^ Silanisation with APTES altered the zeta potential of titania nanosheets *via* amino-end groups, in which the APTES–titania nanosheets have the point of zero charge at pH 6 and it is stable in acidic solution (pH < 4).^128^ Similar to titanate nanotubes which have a lot of hydroxyl groups on its surface, the chemically exfoliated titania nanosheets are also highly reactive to H_2_O_2_. Reaction with H_2_O_2_ creates titanium(iv) peroxo-complex, which is indicated by a colour transformation from white to yellow. Interestingly, the colour reverts back to white after reacting with azo dyes, indicating the release of the oxo group while cutting the azo dye chain.^135^ The colour transformation does not occur in sol–gel titania nanosheets, which is probably due to the hindrance caused by the excess of bulky molecules of TMAOH. Further study is required for the formation of peroxo complexes in sol–gel titania nanosheets.

## 4. Applications

### 4.1. Exposed facets titania

In the past several years, the exposure of certain crystal facets in TiO_2_ has emerged as a highly promising avenue for solving several challenges that hampers the efficiency of conventional TiO_2_ in photocatalysis. The exposure of an unusual active crystal facet in TiO_2_ has garnered significant attention as one of many potential solutions to enhance the photocatalytic performance by improving the light absorption and charge carrier recombination. For example, Wu and co-workers demonstrated that synthesizing rutile TiO_2_ with a tunable ratio of the {110} and {111} facets was evidently able to enhance the photocatalytic activity in the hydrogen evolution reaction.^136^ A tunable ratio of both unusual facets was achieved by using seed-mediated hydrothermal method using NaF as a crystal directing agent. Based on the result, rutile TiO_2_ with wholly {111} facet photocatalyst was found to exhibit the most superior photocatalytic activity towards hydrogen production under the irradiation of UV light. This was attributed to the exposure of the more reactive {111} facet.

In another report, it was shown that exposing the (001) facet in anatase TiO_2_ was also evidently able to provide a significant increase in the photocatalytic activity of the Au–TiO_2_ nanocomposite in the photodegradation of a potent organic dye under visible light.^33^ According to this work, it is evident that anatase TiO_2_ with nanospindle morphology exhibited a four-time higher photocatalytic reaction rate than TiO_2_ with the nanocube morphology. Such enhancement in the activity of the TiO_2_ nanospindles was believed to be due to the high exposure of the (001) facet, which is responsible for improving the migration and separation of the generated charge carriers. As a result, this would allow an efficient prevention of fast electron–hole recombination and lead to a better photocatalytic performance. Similar enhancements in activity for the photocatalytic activity was also observed when the (001) exposed TiO_2_ was composited with other materials, such as two-dimensional graphene oxides or CdSe quantum dots nanoparticles.^67,137^

Recently, a composite of BiVO_4_ and anatase TiO_2_ with co-exposed (001) and (101) facets was also used as photoanode materials, and exhibited good performance in a photocatalytic fuel cell (PFC).^138^ In this study, the as-prepared photoanode was able to exhibit a considerably high photoelectrochemical response with a current density of 29.8 μA cm^−2^ (at 0.8 V *vs.* NHE) under the low-intensity illumination of 13 W LED light. Additionally, the photoanode was able to generate electric power (0.00232 mW cm^−2^) using rhodamine B (RhB) as fuel. It is believed that such enhancement originated from the ability of the co-exposed (001) and (101) facets in TiO_2_ to form an internal surface heterojunction, in addition to the already existing external interfacial heterojunction between BiVO_2_ and TiO_2_. As a result, this would allow a further enhancement and efficient distribution of photogenerated charge carriers.

The exposure of the unusual crystal facet in TiO_2_ has also attracted considerable attention in recent years for the application of solar energy harvesting, particularly in photovoltaic solar cells. Typically, a solar cell relies primarily upon efficient light absorption, charge separation, and transport to maximize the energy conversion efficiency. To serve such purposes, mesoporous semiconducting materials such as TiO_2_ are often used as both support light absorption layer and ETL. Nevertheless, commercial and conventional TiO_2_ often suffer from poor conductivity, inefficient electron mobility, and low diffusion rate of the carrier, leading to low power conversion efficiency. Crystal facet engineering in TiO_2_ presents an intriguing avenue for enhancing the performance of solar cells. The recent surge in research elucidates the potential of exposing certain facets in TiO_2_ to revolutionize solar cell technology through their exceptional properties. For instance, Qaid *et al.* reported that TiO_2_ nanocrystals with exposed {001} facet prepared with facile HF- and NaF-mediated hydrothermal method exhibited a significant improvement in the performance for DSSC.^139^ Additionally, a similar enhancement in performance was observed when the {001} facet-dominant TiO_2_ nanoparticles were used as ETL in CH_3_NH_3_PbI_3_ perovskite solar cells.^140^ According to the experiment, it is evident that the exposure of TiO_2_'s {001} facet was responsible for the enhancement of the electron injection and suppression of electron–hole recombination, which resulted in an increase of both photocurrent and open-circuit voltage.

The application of exposed facet titania within the field of energy storage has emerged as an exciting frontier over the past several years. Energy storage technologies, such as lithium-ion batteries and supercapacitors, play a crucial role in achieving efficient energy utilization and management. Recently, many reports have also highlighted that the exposure of the unusual crystal plane in TiO_2_, characterized by its unique atomic arrangement and distinctive surface properties, has proven to evidently improve the efficiency, stability, and overall performance of energy storage devices. For example, a composite of hierarchically porous TiO_2_ nanosheet with large exposure of the (001) facet and rGO was able to exhibit a superior and stable lithium storage capacity and high performance as an anode material in lithium ion batteries.^141^ Based on the result, it is reported that the anode material showed an excellent reversible capacity of 250 mA h g^−1^ in a voltage window of 1.0–3.0 V and demonstrated good stability even after 1000 cycles. In another report, Wang and co-workers compared the performance of the (001)-faceted TiO_2_ nanosheet *vs.* spherical TiO_2_ nanoparticles as anode material in lithium ion batteries.^142^ Here, it is evident that the battery fabricated with the (001)-faceted TiO_2_ nanosheet exhibited superior storage capacity, enhanced stability, and higher charge/discharge rate compared to that of the spherical TiO_2_ nanoparticles. It is believed that such enhancement was due to the ability of the exposed (001) facet in TiO_2_ to facilitate an efficient charge diffusion, which led to an increase in the rate of Li ion insertion/extraction along the *c*-axis during the charge–discharge.

### 4.2. Monolayer titanate

Most monolayer titania nanosheets are made by top-down approaches through the exfoliation of layered titanate compounds. The layered structured of titania also has many applications. Having a layered structure, the interlayer cations can be reversibly exchanged with other cations. The ion exchange properties enable the layered nanosheets to adsorb radioactive ions; hence, it is useful for environmental remediation. Several researchers utilised acid-modified titania nanosheets for Cs^+^ ion adsorption, in which the adsorption capacity did not decrease even after 5 cycles.^143^ The adsorption capacity of Cs^+^ ions reached 329 mg g^−1^, which is promising for radioactive wastewater treatment. Protonated TiNS was also able to adsorb cationic dyes such as methylene blue with the adsorption capacity up to 3937 mg g^−1^, following the Langmuir model.^144^ For dye removal, peroxo-modification of the TiNS surface could be done, changing the colour of titania from white to yellow.^135^ With hydrogen peroxide, the Ti(iv)–H_2_O_2_ complex was formed, creating TiOOH moieties on the surface. The peroxo groups were then able to oxidise dyes into smaller molecules. Hence, the dye removal can be performed without the assistance of UV or visible light. The interlayer spacing and surface charge of acid-modified TiNS also induced size selectivity for adsorbing the pharmaceutical compound, fluoroquinolone.^145^ In neutral and acidic solutions, the acid-modified TiNS was able to be intercalated by positively CIP with a thickness of 0.41 nm. Selective adsorption was also obtainable by surface modification of TiNS.^128,146^ Boronic acid ligands were immobilized on the surface of modified TiNS, resulting in the selective adsorption of IgG up to 1669.7 mg g^−1^ capacity.^146^ APTES-modified TiNS was deployed a as nanocontainer of DNA.^128^ The DNA was intercalated in the layer of APTES–TiNS, where it was protected by TiNS from enzymatic corrosion, acid condition, and UV-vis light irradiation. Thus, DNA could be stored and released on demand.

The ion exchange capacity of TiNS can also facilitate energy storage. During charging and discharging, the intercalation and de-intercalation of cations occur. Layered titania nanosheets with minimal layer-to-layer interaction and a robust gallery space enabled the fast and stable intercalation and de-intercalation of large ions such as sodium and potassium ions in a non-aqueous electrolyte.^147^ To obtain the minimum layer-to-layer interaction, the titania nanosheets were exfoliated *via* a chemical exfoliation method, followed by coagulation with a Mg^2+^ solution to obtain a randomly stacked nanosheet structure. At a rate of 3000 mA g^−1^, the capacity was retained at more than 80% after 10 000 cycles for Na^+^ ion storage, which was performed using an electrode thickness of 80 μm. Such remarkable performances did not occur without the prior exfoliation of titanates. The specific capacity for Na^+^ ion storage was 53 mA h g^−1^ and 188 mA h g^−1^ without and with prior exfoliation, respectively. Nevertheless, the theoretical capacity of titanate is relatively small compared to that of graphite or SnO_2_.^8^ Doeff *et al.* synthesised the composite of carbon–TiNS by exfoliating the titanate structure, followed by carbonization of dopamine for the sodium half-cell configuration.^148^ The hetero-structure of carbon-titania resulted in higher capacity and capacity retention, while lowering the impedance. The combination of titania nanosheets with SnO_2_ for the sodium ion battery should be expected in the near future. The titania nanosheets could also be used as an electrode for electroanalysis.^121,130,149^ The titania nanosheet exfoliated with tetrabutylammonium cations was deposited from a colloidal aqueous solution onto a glassy carbon electrode, creating a lamellar structure.^130^ The lamellar titania acted as a sorbent and host for the hydrophobic redox system and for electrochemical reactivity. A future study on the electron transfer, mobility, and binding of guest species within the lamellar is intriguing. The negatively charged TiNS could also act as a host of ferroceneboronic acid receptor molecules, exhibiting the selective sensing of fructose while insensitive for glucose.^149^ Moreover, the cationic diode behaviour was observed using the TiNS deposit on top of the micron-sized hole of the PET film.^150^ The ionic current rectification was possible due to the negative surface charge of TiNS and tortuous path of ions within the lamellar space.

Titanium dioxide is known to show striking photocatalytic activities, while the high surface area of the 2D nanosheets increases the density of active sites. TiNS has a larger band gap (*i.e.*, 3.84 eV) than anatase TiO_2_ (3.2 eV).^120^ A strong UV light is needed to excite the electrons for photocatalysis. Therefore, many researchers combine TiNS with other catalysts to obtain a narrow band gap, while maintaining the high surface area. One group of researchers combined positively charged Zr-EDTA complexes with negatively charged TiNS, creating a porous structure with a surface area of 193 m^2^ g^−1^ and a specific pore volume of 0.39 mL g^−1^.^151^ The composite of Zr-EDTA–TiNS yielded a band gap of 3.15 eV and was used for degrading methylene blue (MB) under UV irradiation. The photocatalytic degradation kinetics of methylene blue was 5-fold higher and reached 98.1% MB removal for the Zr-EDTA–TiNS composite, as compared to TiNS alone. The photocatalytic mechanism can be described as an artificial Z-scheme heterostructure due to ohmic contact, facilitating charge transfer between the conduction band of TiNS and valence band of Zr-EDTA. TiNS has also been combined with alkaline Co(OH)_2_ (ref. 152) and Ni(OH)_2_ (ref. 153) for the photocatalytic reduction of CO_2_. The alkaline Co(OH)_2_ and Ni(OH)_2_ acted as a CO_2_ binder, while TiNS adsorbed the sensitiser and became an electron relay that bridged the sensitiser with Co(OH)_2_ and Ni(OH)_2_ active sites. For Ni(OH)_2_–TiNS, the production rate of CO/H_2_ was 1801/2093 μmol g^−1^ h^−1^, while Co(OH)_2_–TiNS was 56.5/59.3 μmol h^−1^. For photovoltaic application, TiNS was used as an atomic stacking transporting layer (ASTL) in the lead halide perovskite solar cell.^154^ The TiNS was stacked into a multilayer thin film by layer-by-layer deposition, which achieved complete surface coverage after 5 repetitions. Contrary to the conventional sintered TiO_2_ thin film, the layer-by-layer deposition of TiNS exhibited nearly negligible oxygen vacancies. The oxygen vacancies may cause UV instability of the perovskite solar cell. For titania nanosheets ASTL, the power conversion efficiency remained at around 70% of the initial value after 5 hours of UV irradiation, while severe reductions of PCE occurred for the conventional TiO_2_ thin film, resulting in only 5% initial value of PCE. Besides photovoltaic application, TiNS could also be used for hydrovoltaic devices.^155^ The electricity was generated from water evaporation. The titanium vacancy of TiNS enhanced the water–solid interaction. When water molecules flow over the solid surface, the migration of counterions occurs to generate an electric output. The hydrovoltaic device based on TiNS produced an open circuit voltage of 1.32 V for more than 250 h.

Coatings of layer-by-layer deposition of TiNS were used to protect stainless steel car baffle from corrosion.^156^ The five-cycle layer-by-layer deposition of TiNS exhibited a thickness of around 10 nm with a corrosion inhibition efficiency of 99.92% and an estimated corrosion rate of 5.32 × 10^−5^ mm per year. The 2D structure of TiNS created a tortuous path for iron and oxygen diffusion, hampering the rusting process of iron. Titania nanosheets have been known as a strong adsorbent of rare earth elements, such as Eu, exhibiting photoluminescence properties.^157^ Intense red emission was observed at 616 nm under the irradiation of 400 nm UV LED light. It would be interesting to combine layers of red-emitting TiNS with blue-emitting rare-earth mixed metal oxides, such as BaMgAl_11_O_17_: Eu^2+^ (ref. 158) to create multi-colour luminescent layers for monitoring coating health. As a nanocomposite coating, silk–TiNS enhanced the tribological properties (*e.g.*, hardness, reduced modulus, wear, adhesion, and scratch resistance) of silk coatings.^159^ The hardness and reduced modulus of the silk–TiNS composite were higher than those of the graphene–silk composite film. The reinforcement behaviour also occurred for bulk polymer nanocomposites, following micromechanical models such as Halpin–Tsai and Brune–Bicerano, up to few number of nanosheets layers.^160^ As discussed in section 3.1.2, TiNS is sensitive to magnetic flux and UV light, in which the orientation of TiNS within a polymer matrix can be adjusted. Hence, stimuli-responsive polymer nanocomposites could be realised by incorporating TiNS within the polymer. A silk–TiNS multilayer thin film also exhibited moisture-responsive coating.^161^ The water molecules were adsorbed into the nanosheets, causing swelling and reduction of the refractive index of the film. In summary, the layered 2D structures of nanosheets and the photoresponsive, chemically stable, negatively charged TiNS have many existing and potential applications worthy of further investigation in combination with other nanomaterials or polymers.

## 5. Conclusions and outlook

In conclusion, two-dimensional TiNS has emerged as a multifaceted and promising material that has captured the attention of the scientific community. With significant implications for fields, ranging from catalysis, electronics, and energy conversion to environmental remediation, energy storage, and biomedical applications, TiNS offers a transformative potential. Key to this aspect is the manipulation of their crystal facets and structures, which allow for a tailored set of properties for diverse applications. Synthesis routes involving both exposed facet and monolayer titania nanosheets have demonstrated unique properties, such as heightened catalytic activity, ion-exchange capabilities, and exceptional optoelectronic behaviours.

Within the realm of synthesis, hydrothermal and solvothermal methods have proven effective for facet control. There has been a shift toward non-fluorine-based precursors, primarily due to the associated safety and environmental considerations. This trend aligns well with the broader scientific movement toward more sustainable and eco-friendly materials. In contrast, the chemical exfoliation methods use non-fluorine precursors, offering a safer yet versatile route to monolayered structures.

For applications, TiNS demonstrates a myriad of functionalities. Their ion-exchange properties make them valuable candidates for environmental applications, such as the absorption of radioactive ions and organic dyes. The adaptability of TiNS in energy storage, particularly sodium and potassium ion batteries, and their potential in photocatalysis, signal an exciting trajectory for these materials. Composite structures have shown that TiNS can work in synergy with other materials to further enhance their performance in these sectors.

As we look to the future, the focus should be on refining and diversifying non-fluorine-based synthesis methods and deepening our understanding of the relationship between the crystal structure and material properties. Exploring hybrid composites, particularly through the integration of TiNS with polymers and other nanomaterials, appears to be a promising avenue. Moreover, targeted research into nanoengineering for optimizing energy storage and tunable band gaps for photocatalytic applications holds significant potential. These endeavours not only serve to advance our technological capabilities, but also usher in an era of increased safety, energy efficiency, and environmental consciousness.

## Abbreviations

[bmim]-[BF_4_]: 1-Butyl-3-methylimidazolium tetrafluoroborate
2D: Two dimensional
3D: Three dimensional
AFM: Atomic force microscopy
APTES: 3-Aminopropyl triethoxysilane
ASTL: Atomic stacking transporting layer
CB: Conduction band
CBM: Conduction band minimum
CIP: Charged ciprofloxacin
DETA: Diethylenetriamine
DFT: Density functional theory
DMF: *N*,*N*-Dimethylformamide
DNA: Deoxyribonucleic acid
DSSC: Dye-sensitized solar cells
EBD: Electron beam deposition
EDTA: Ethylenediaminetetraacetic acid
ETL: Electron-transporting layer
FESEM: Field emission scanning electron microscopy
HF: Hydrofluoric acid
IgG: Immunoglobulin G
ITO: Indium-doped tin oxide
MB: Methylene blue
MO: Methyl orange
MXenes: Two-dimensional transition metal carbide
NTA: Nanotube titanic acid
PET: Poly(ethylene-terephthalate)
PFC: Photocatalytic fuel cell
PVP: Poly(vinylpyrrolidone)
rGO: Reduced graphene oxide
SAXS: Small-angle X-ray scattering
SDS: Sodium dodecyl sulfate
SEM: Scanning electron microscopy
SSA: Specific surface area
TBA: Tetrabutylammonium
TEM: Transmission electron microscopy
TiNS: Titania nanosheets
TIP: Titanium isopropoxide
TMA: Tetramethylammonium
TOB: Truncated octahedral bipyramidal
UV: Ultraviolet
UV-vis: Ultraviolet-visible light
VBM: Valence band maximum
XRD: X-ray diffraction

## Data availability

No primary research results, software or code have been included, and no new data were generated or analysed as part of this review.

## Conflicts of interest

There are no conflicts to declare.
